# A stability-indicating potentiometric platform for assaying Metoprolol succinate and felodipine in their tablets and human plasma

**DOI:** 10.1186/s13065-025-01435-z

**Published:** 2025-03-19

**Authors:** Haitham A. El Fiky, Mahmoud A. Tantawy, Dina A. Ahmed, Maha F. Abd El Ghanyd, Amr M. Badawey, Nermine V. Fares

**Affiliations:** 1 Pharmaceutical Chemistry Department, Faculty of Pharmaceutical Sciences and Pharmaceutical Industries, Future University in Egypt, Cairo, Egypt; 2 Pharmaceutical Analytical Chemistry Department, Faculty of Pharmacy, Cairo University, Kasr El-Aini Street, Cairo, ET-11562 Egypt; 3 Department of Chemistry, Faculty of Pharmacy, October 6 University, 6 of October City, Giza, Egypt; 4 Analytical Chemistry Department, Faculty of Pharmacy, Ain Shams University, Cairo, Egypt

**Keywords:** Metoprolol, Multi-walled carbon nanotubes, Felodipine, Molecular imprinted polymer, Logimax^®^

## Abstract

Solid contact electrodes are prevalent in analytical applications due to their superior performance compared to traditional electrodes. Nonetheless, these electrodes have been observed to develop a water layer, which compromises their stability. In this study, we introduce an innovative solid contact ion selective electrode designed to mitigate this issue by incorporating multi-walled carbon nanotubes. This system was utilized for potentiometric sensing of metoprolol and felodipine. Furthermore, molecular imprinted polymer was developed to enhance selectivity for determination of felodipine. The electrode modified with multi-walled carbon nanotubes was employed for the quantification of metoprolol, exhibiting a Nernstian slope of 55.23 mV/decade over a linear concentration range of 1.0 × 10^− 7^ to 1.0 × 10^− 2^ mol L^− 1^, at a pH of 7.0. The molecularly imprinted polymer-modified electrode was utilized for the determination of felodipine, showing slope of 56.089 mV/decade across a linear range of 1.0 × 10^− 7^ to 1.0 × 10^− 4^ mol L^− 1^, at a pH of 3.0. Detection limits for both sensor were less than 8.0 × 10^− 8^ mol L^− 1^. The developed sensors were successfully utilized for the quantification of the aforementioned drugs in pharmaceutical tablets, in human plasma samples and in the presence of their degradates. The proposed approach showed a better linearity range and a lower limit of detection for metoprolol quantification compared to its reported potentiometric methods. Moreover, it was the first one to use such an electrochemical technique for felodipine detection.

**Supplementary Information:**

The online version contains supplementary material available at 10.1186/s13065-025-01435-z.

## Introduction

Molecularly imprinted polymers (MIPs) and solid contact ion-selective electrodes (SC-ISEs) are two distinct and advanced technologies that have significantly enhanced the performance of electrochemical sensors. In the development of potentiometric sensors, MIPs are used to create highly selective binding sites tailored to a specific target molecule, mimicking the shape and functional groups of that molecule [1–2]. These MIP-based sensors are particularly effective in detecting specific drugs, where the polymer’s selectivity allows for precise discrimination between the target analyte and interfering substances in complex matrices. The incorporation of MIPs into SC-ISEs enhances the sensor’s ability to selectively bind the drug of interest, providing reliable and accurate measurements in applications such as pharmaceutical analysis and clinical diagnostics [3–4].

On the other hand, carbon nanotubes (CNTs), particularly multi-walled carbon nanotubes (MWCNTs), have been employed in SC-ISEs for a different set of advantages. MWCNTs are used to enhance the electrical conductivity and surface area of the electrode, which are critical for efficient ion-to-electron transduction. In sensors designed for another specific drug, the integration of MWCNTs into the solid contact layer improves the electrode’s stability, response time, and overall sensitivity [5]. The hydrophobic nature of MWCNTs also helps to minimize the formation of an unwanted water layer at the interface between the sensing membrane and the solid contact, which is a common issue in traditional electrodes. This leads to more stable and reliable measurements, particularly over extended periods, making MWCNT-based SC-ISEs suitable for continuous monitoring and long-term applications [6].

The combination of MIPs and MWCNT-based SC-ISEs holds significant promise for developing highly sensitive and selective potentiometric sensors. The customizable nature of MIPs allows for the design of sensors tailored to specific analytes, while the integration of MWCNTs enhances the overall performance of the electrode, particularly in terms of stability and response time. This synergy between MIPs and MWCNTs could lead to the development of next-generation sensors capable of operating in a wide range of clinical settings, offering both high performance and reliability [7, 8].

Metoprolol Succinate (MET); Fig. S1.a is chemically known as bis [(2RS) -1-[4-(2-methoxyethyl) phenoxy)-3-[(1methylethylaminopropan-z-ol] butanedioate. MET is a beta- 1 receptor blocker that works to lower blood pressure by reducing the pumping force of the heart and thus reduces the amount of blood pumped into the blood vessels. The drug is 90% metabolized *via* hydroxylation and O-demethylation [9, 10]. It has pka ≈ 9.1 [11] so it will be ionized and have positive charge at pH lower than 8.0.

Felodipine (FEL); Fig. S1.b is chemically designed as ethyl methyl (4RS)-4-(2, 3-dichlorophenyl)-2,6-dimethyl-1,4dihydropyridine-3, 5-dicarboxylate. It belongs to a group of medicines that prevent the passage of calcium, as calcium works to constrict blood vessels, and therefore the lack of calcium leads to widening of blood vessels and thus works to reduce high blood pressure. It is completely metabolized as no unchanged drug is excreted in urine [12, 13]. It has pka ≈ 5.1 [14] so it will be ionized and have positive charge at pH 2.0–4.0.

Logimax^®^ tablets, comprising a binary combination of MET and FEL, have garnered a widespread indication for treating hypertension. The synergistic mechanisms of action between the two substances result in a more pronounced antihypertensive effect compared to either drug in monotherapy, showcasing the efficacy of this combination for effective blood pressure management.

Few analytical spectrophotometric [15, 16], spectrofluorometric [17], HPTLC [18–20] and HPLC techniques [21–24] for analyzing MET and FEL have been stated after examination and scrutiny of previous research. In the meantime, there is no potentiometric method for determination of their combination, only few potentiometric methods were reported for determination of MET [25–28]. Regarding FEL, there is no reported potentiometric method. Therefore, our aim is to propose two selective electrodes for potentiometric assessment of MET and FEL in their combined dosage form, human plasma and in-presence of their degradates. The proposed sensor demonstrated selective determination of the target analytes without interference from other substances. Carbon paste electrode modified with MWCNTs and positioned as an interlayer, were employed to enhance the reproducibility and stability of the solid contact ion selective electrodes (SC-ISEs). A molecular imprinted polymer was incorporated in FEL sensing membrane to enhance its selectivity in presence of the positively charged interfering MET.

## Experimental

### Materials and reagents

The studied drugs’ pure samples were kindly acquired from Global Napi, Egypt. According to MET’s and FEL’s official methods [9, 12], the stated purities were found to be 99.55 ± 0.65 and 99.65 ± 0.98, respectively. The Logimax^®^ tablet, produced in England by Astrazeneca, was acquired for the market. The batch (210505) of Logimax^®^ includes 50 mg of metoprolol succinate and 5.0 mg of felodipine.

All used chemicals and reagents were of analytical grade. Azobisisobutyronitrile (AIBN), ammonium persulfate (APS), dimethylsulfoxide (DMSO), chloroform, aniline, polyvinyl chloride (PVC), sodium dodecyl sulfate (SDS), toluene, multiwall carbon nanotubes (MWCNTs; ≥ 98% carbon basis), methanol, ethylene glycol dimethacrylate (EGDMA), tetrahydrofuran (THF), glacial acetic acid, ethanol, methacrylic acid (MAA), graphite powder (20 mm), potassium tetrakis (4-chlorophenyl) borate (TpClPB), 2-nitrophenyl octyl ether (NPOE), 30% H_2_O_2_ and paraffin oil were all obtained from Sigma-Aldrich (Germany). A Britton-Robinson buffer (BRB) was obtained by combining 40 mM acetic acid, 40 mM boric acid and 40 mM phosphoric acid, then adjusting the pH to the required range of 2.0–9.0 using 0.2 M NaOH. Blank Human plasma was supplied from the Holding Company for Biological Products and Vaccines (VACSERA, Giza, Egypt).

### Instrumentation

- Ag/AgCl reference electrode (Thermo Scientific, USA).
- pH glass electrode and pH meter model 3510 (Jenway, UK).
- Carbon paste electrode working electrode model MF-2010 (BASi, USA).
- A water bath model WB-22 (DAIHAN Scientific, South Korea).
- IR Spectrophotometer model 435 (Shimadzu, Japan).
- Differential scanning calorimeter DSC-60 (Shimadzu, Japan).
- Field emission scanning electronic microscope model FEG (Oregon, USA).
- Soxhlet apparatus (MXBAOHENG, USA).

### Molecular imprinted polymer Preparation for FEL

In order to create MIPs, this study employed the non-covalent precipitation polymerization method [29, 30]. This approach was selected to obtain regular-sized and shaped particles with low electrical impedance [2]. The ratio between the template FEL and MAA was also investigated and the 1:4 ratio was selected based on the reported fabrication processes utilized in electroanalysis [2]. Therefore, to 1 mmol of FEL, 40 ml of DMSO (a porogenic solvent) was put in a glass-stoppered measuring flask. The pre-polymerization complex was allowed to self-assemble by adding 4 mmol of MAA to this solution and subjecting it to sonication for 15 min. Then, 1 mmol of AIBN, an initiator, and 25 mmol of EGDMA, a cross-linker, were added. Polymerization was permitted to take place in a thermostatic water bath at 60 °C for twenty-four hours after the flask was sprayed with nitrogen for around ten minutes. To remove any unreacted components, the white precipitate was filtered after two ethanol washes, a 15-minute shaking, and a decantation filter. The MIP particles were treated with a 9:1 v/v mixture of methanol and glacial acetic acid to remove the template. This was accomplished by batch-mode Soxhlet extraction. Until the extract solution no longer exhibited drug absorbance, the extraction process was monitored using UV/Vis measurements before being dried at 100 °C in an oven. Distilled water was used to wash MIP until it attained a neutral pH. In order to make the corresponding non imprinted polymer (NIP) for FEL, we followed the steps outlined earlier but omitted the template. In this case, FEL-MIP denoted the prepared MIP, while FEL-NIP denoted the NIP for FEL. Both MIP and NIP had their morphology thoroughly examined using field-emission surface electron microscopy (FE-SEM) and differential scanning calorimetry (DSC). In addition, UV spectrophotometry was employed to monitor the re-binding capability of the generated MIPs and determine their binding capacity.

### Sensors fabrication

An evenly moist paste was produced by mixing 4.3 g of paraffin oil, 2 g of MWCNT, and 8 g of graphite powder in an agate mortar for 30 min [31]. This was done to make the carbon paste that was utilized. Polishing the paste against filter paper after pressing it firmly into the CPE chamber produced a shiny surface. In the end, The ion-sensing membrane was fabricated following the reported procedures [2, 8] by applying two drop-casts of 10 µl of a mixture composed of 200 milligrams of PVC, 0.4 milliliters of NPOE, 1 milligram of TpClPB, and 10 milligrams of the sensing polymer (either FEL-MIP or FEL-NIP) dissolved in 5 milliliters of tetrahydrofuran.

The MET-CPE electrode was constructed according to the previously specified processes, except that no MIP was added. Fig. S2 shows a photo of the proposed potentiometric system illustrating the utilized carbon paste composites along with the sensing membranes’ components.

After allowing each electrode to air dry, they were submerged in the appropriate analyte solutions of 1 × 10^− 4^ M in buffer pH 7.0 for MET and 1 × 10^− 4^ M in buffer pH 3.0 for FEL for a full day.

### Potentiometric measurement

With BRB that had been adjusted to pH 7.0 and pH 3.0 respectively, separate stock solutions of MET (1 × 10^− 2^ M) and FEL (1 × 10^− 2^ M) were prepared. Two of 25 ml volumetric flasks were subsequently diluted using the buffer, resulting in 1 × 10^− 7^ M − 1 × 10^− 2^ M for MET and 1 × 10^− 7^ M − 1 × 10^− 4^ M for FEL as concentration ranges. Potentiometric measurements were conducted using MET/MWCNTS and FEL-MIP/MWCNTS sensors. We computed the regression equations by taking potentiometric readings at various drug concentrations using the suggested sensors.

### Degradate samples

For eight hours at 80 °C, a round flask containing known amount of MET and 10 ml of 30% H_2_O_2_ was refluxed. The oxidatively degraded product was obtained after hydrogen peroxide was evaporated using a hot plate set at 50 °C, then transferred to a volumetric flask containing 100 ml of BRB that had been pH-7.0 adjusted. In a separate round flask, a known amount of FEL was refluxed with 10 milliliters of 3 N HCl for eight hours at 80 °C. The refluxing process was followed by the addition of 3 N NaOH to neutralize the solution. The acid degradation product was then transferred to a 100 ml volumetric flask and the solution was completed with pH 3.0 adjusted BRB. Each degradation product was produced at a concentration of 1 × 10^− 4^ M of the corresponding intact drug.

### Application to pharmaceutical formulation

Ten Logimax^®^ tablets were powdered. Amount equivalent to 50 mg of MET and 10 mg FEL were moved to different 25-ml volumetric flasks and adjusted with buffer solution of pH 7.0 and pH 3.0 to prepare stock solution of 3.0 × 10^− 3^ M of MET and 1 × 10^− 3^ M of FEL, respectively. Potentiometric measurement of the solutions was conducted by submerging the two suggested sensors in Ag/AgCl reference electrode. Each drug’s concentration was calculated using the corresponding regression equation.

### Application to spiked human plasma

From the MET and FEL standard solutions, 1 mL and 0.5 mL aliquots were respectively transferred to separate 10-ml volumetric flasks. Then, 2 ml of human plasma was added and diluted to the correct volume using pH adjusted BRB. The two proposed sensors were used for potentiometric measurement of the solutions after one minute of sonication.

## Results and discussion

For the analysis of various lipophilic drugs, ISEs have been developed to function in both positive and negative modes, enabling the detection of a wide range of substances [32, 33]. However, determining the potentiometric response of two lipophilic drugs that share the same ionic charge presents a significant analytical challenge. This difficulty arises from the sensing membrane’s limited capacity to differentiate between compounds with similar lipophilicity and electrical charges. In such scenarios, the doped ion exchanger within the membrane does not effectively discriminate between different moieties that possess comparable physicochemical properties, thereby complicating the simultaneous detection and accurate measurement of these drugs [34].

Regarding MET, it has logP = 1.76 [35] and it is positively ionized at pH lower than 8.0, while FEL has logP = 3.86 [13] and it is positively ionized from pH 2.0–4.0 [36] so there will be no interference from FEL at pH 7.0 as it will be non-ionized. The challenge here was for determination of FEL in-presence of MET without any interference so MIP was developed for determination of FEL to enhance selectivity.

Water layer formation between the membrane and solid contact ion selective electrodes (SC-ISEs) is the primary challenge that affects the stability and reproducibility of SC-ISEs, as well as the composition of the sample. Currently, this obstacle can be circumvented by the incorporation of carbon nanotubes (CNTs), therefore the production and doping of carbon paste with MWCNT as a hydrophobic layer to overcome formation of water layer. The goal of the second stage was to overcome the selectivity barrier by synthesizing, thoroughly characterizing, and integrating MIPs for FEL into the appropriate sensing membrane thereby allowing the potentiometric determination of the drugs under study in the presence of one another by effectively utilizing the high recognition binding capability of MIP. The sensors that were developed, namely MET/MWCNTS and FEL-MIP/MWCNTS, were utilized for the analysis of the pharmaceuticals being investigated. These sensors were employed not only to detect the presence of the pharmaceuticals, but also the ability to accurately determine the target analytes not only in the binary mixture but also in plasma solution and in the presence of their degradation products.

### Characterization of MIP

**Fig. 1:**
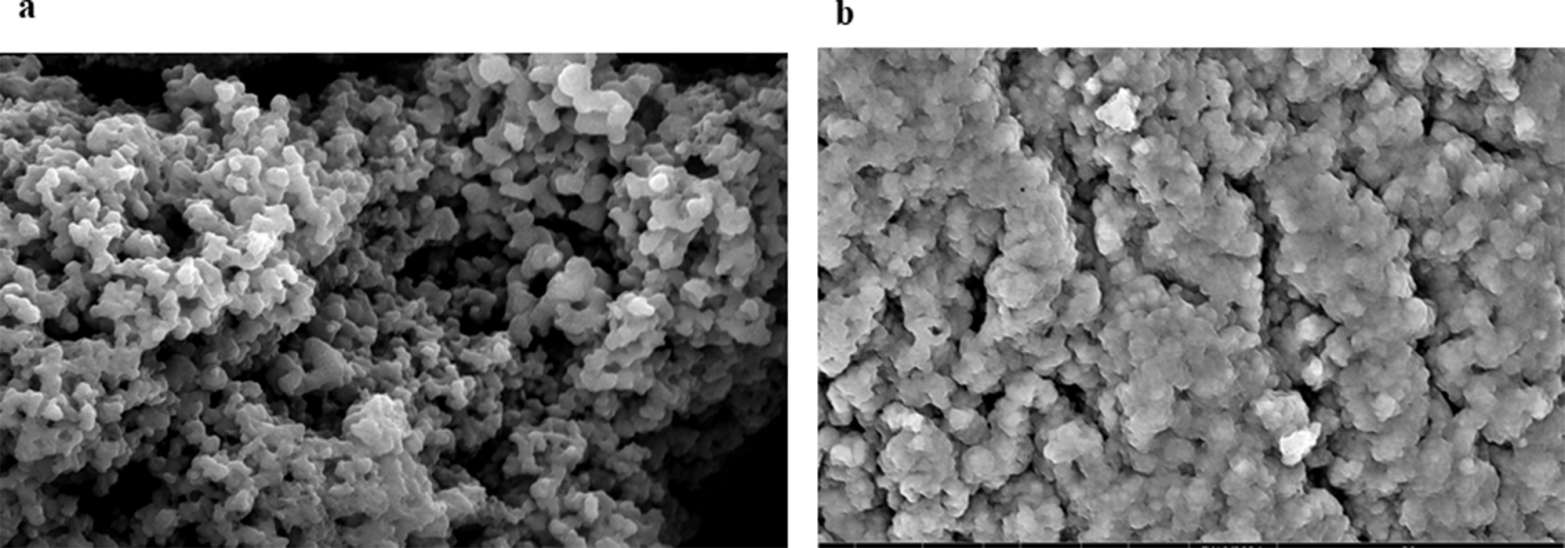
SEM images for the prepared MIP of FEL (**a**) and its corresponding NIP (**b**) at the magnification power of 10^4^

The surface of the leached MIP and NIP prepared for felodipine were examined using SEM. The results presented in Fig. 1 indicate that MIP exhibits a more porous and rough structure in comparison to the plain surface of NIP. This observation suggests the presence of small voids that are created by the removal of the drug from the template during the synthesis process. It is worth noting that this porous structure resembles a “lock” complementary in shape and size to the “key” drug, thus enhancing the electrode’s selectivity.

**Fig. 2:**
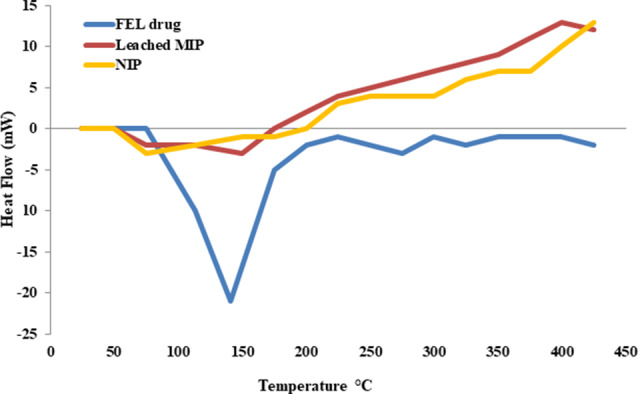
DSC thermograms for FEL along with its corresponding leached MIP and NIP

By recording DSC thermograms for pure FEL with its MIP and NIP particles, Fig. 2 showed endothermic peak at 141 °C which corresponds to its relative melting temperature [37]. The previously mentioned endothermic peak of the drug was absent in the accompanying leached MIP provided additional evidence of the excellent removal of the template, as indicated by DSC. Figure 2 also showed absence of FEL peak for MIP and NIP.

One of the potential limitations of utilizing MIP in sensing applications is the presence of non-specific binding sites. As a result, the rebinding capacity of MIP-FEL was assessed to guarantee its effectiveness. 20 mg of FEL-MIP or FEL-NIP was added to a 0.05 mM FEL solution in buffer adjusted to pH 3.0. After then, the UV absorbance at 238.0 nm was noted.

Evaluation of the prepared MIP rebinding capacity was achieved according to the following equation $\:Q=\frac{\left({C}_{i}-{C}_{f}\right)\times\:V\times\:1000}{{M}_{Polymer}}$

Q is the binding capacity, V is the prepared solution volume (ml), M_polymer_ is the mass of added polymer (mg), and C_i_ and C_f_ are the starting and remaining drug concentrations (mM), respectively. Binding capacity (Q) of MIP and NIP are 52 and 25 µmol g^− 1^, respectively. The obtained results showed the higher binding capacity of MIP compared to NIP, indicating higher selectivity as well as neglected non-specific adsorption in the prepared MIP.

### Potentiometric water layer and potential stability tests

**Fig. 3:**
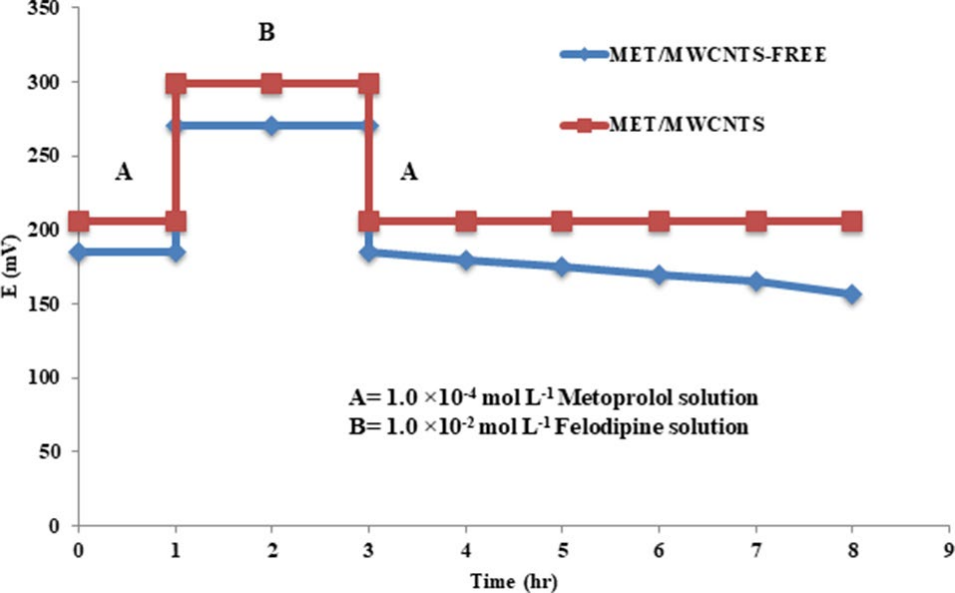
Water layer test for MET/MWCNTS and MET/MWCNTS-Free electrodes where potential in (mV) was recorded in (**A**) 1.0 × 10^− 4^ mol L^− 1^ metoprolol solution and (**B**) 1.0 × 10^− 2^ mol L^− 1^ felodipine solution

The creation of a thin aqueous layer at the interface between the solid contact and the ion-sensitive membrane has a detrimental impact on the found electrical signal. The existence and amount of a water layer were evaluated and measured through a water layer test [38]. The test relies on detecting any change in the potential when transitioning from a solution of MET (1.0 × 10^− 4^ mol L^− 1^) to a higher concentration solution of the interfering ion (1.0 × 10^− 2^ mol L^− 1^ felodipine), and then returning to the MET solution. The identification of any possible shifts indicates the formation of a water layer, as illustrated in Fig. 3. Conversely, substantial drifts were detected for the MET/MWCNTS-Free electrode due to changes in the ionic composition of the water layer [38]. In nutshell, the integration of MWCNTs into the proposed ion-selective membrane enhanced its hydrophobicity.

The long-term potential stability of both the MET/MWCNTS and MET/MWCNTS-Free sensors was also assessed. Fig. S3 illustrates a potential deviation of 0.6 mV h^− 1^ for the MET/MWCNTS sensor, which increases to 5 mV h^− 1^ in the case of the MET/MWCNTS-Free electrode after over ≈ 12 h for a 1.0 × 10^− 4^ mol L^− 1^ solution of metoprolol.

### Performance characteristics of the CPEs

**Fig. 4:**
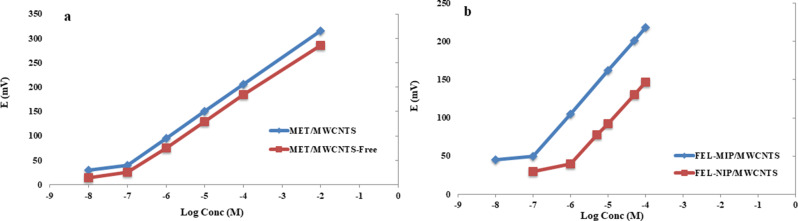
Potential profile to the logarithm of concentrations of (**a**) metoprolol using MET/MWCNTS and MET/MWCNTS-Free electrodes and (**b**) felodipine using FEL-MIP/MWCNTS and FEL-NIP/MWCNTS electrodes

The performance characteristics of MET/MWCNTS and FEL-MIP/MWCNTS, MET/MWCNTS-Free and FEL-NIP/MWCNTS are illustrated in, Fig. 4; Table 1. The obtained results showed low LOD values for both MET/MWCNTS and FEL-MIP/MWCNTS compared to their counter electrodes. These achieved LODs approach the reported maximum plasma concentrations of the studied drugs (in the range of ≈ 10^− 8^ mol L^− 1^), thus suggesting their feasible applications in biological fluids.

**Table 1:** Electrochemical response characteristics of the proposed electrodes

Parameter	MET/MWCNTS electrode	MET/MWCNTS-Free electrode	FEL-MIP/MWCNTS electrode	FEL-NIP/MWCNTS electrode
Slope (mV/decade)	55.230	52.365	56.089	48.400
Intercept (mV)	426.70	391.35	381.80	325.00
Range (mol L^− 1^)	1.0 × 10^− 7^– 1.0 × 10^− 2^	1.0 × 10^− 7^– 1.0 × 10^− 2^	1.0 × 10^− 7^– 1.0 × 10^− 4^	1.0 × 10^− 6^– 1.0 × 10^− 4^
LOD (mol L^− 1^) ^a^	5.0 × 10^− 8^	8.0 × 10^− 8^	7.0 × 10^− 8^	9.0 × 10^− 7^
Working pH	3.0–8.0	3.0–8.0	2.0–4.0	2.0–4.0
Response time (s)	5	8	5	8
Stability (days)	45	35	45	35
Correlation coefficient (r)	0.9999	0.9996	0.9999	0.9996
Accuracy ^b^	99.97 ± 1.48	-	100.67 ± 0.46	-
Precision:				
Repeatability ^c^	0.887	-	1.071	-
Intermediate precision ^c^	1.423	-	1.678	-
Reproducibility ^d^	1.737	-	1.4	-

^a^ Limit of detection (as per the IUPAC definition, measured by interception of the extrapolated arms of non-responsive and the Nernstian segments of the calibration plot

^b^ Mean ± RSD% of recoveries for five concentration levels measured in triplicate

^c^ RSD% of recoveries for three concentrations of MET (1 × 10^− 6^, 1 × 10^− 5^ and 1 × 10^− 4^ M) and of FEL (1 × 10^− 6^, 1 × 10^− 5^ and 5 × 10^− 5^ M), each repeated three times within the day for repeatability and repeated in three successive days for intermediate precision

^d^ RSD% of recoveries for two concentrations (1 × 10^− 5^ M and 1 × 10^− 4^ M) measured using three batches of each CPE

### Influence of various experimental factors

#### pH effect

**Fig. 5:**
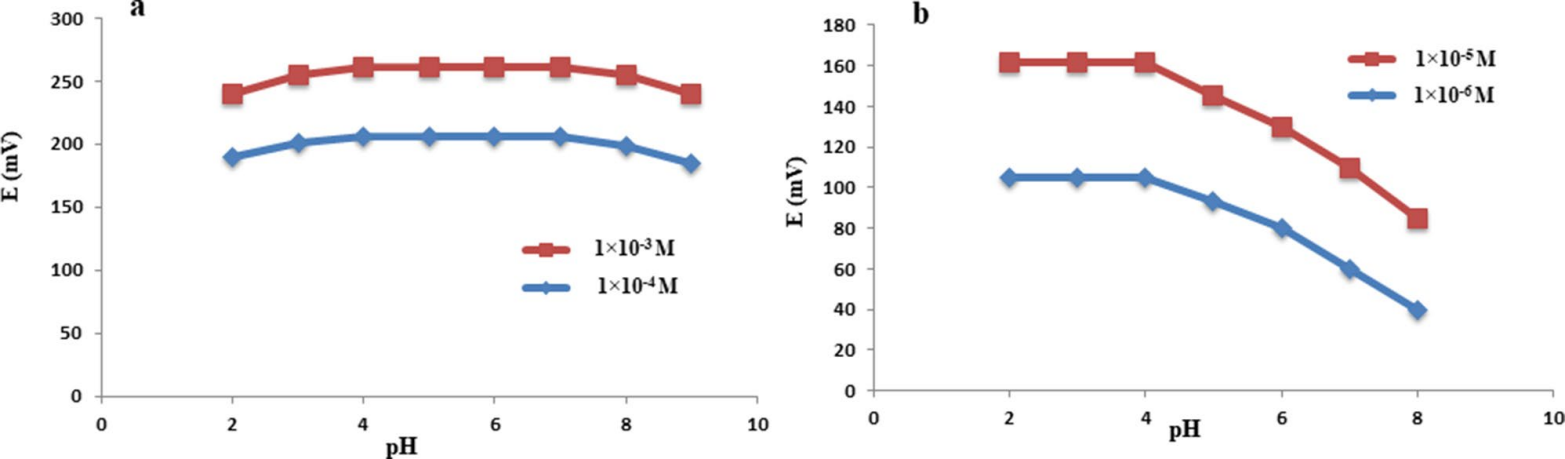
pH effect on response of (**a**) MET/MWCNTS and (**b**) FEL-MIP/MWCNTS electrodes

To study pH effect, the potential readings of two concentrations of MET, and FEL, over a pH range of 2.0 to 9.0 was monitored, as shown in Fig. 5. For MET and FEL, a comparatively consistent potential was found over the pH range of 3.0–8.0 and 2.0–4.0, respectively. The observed potentials, in contrast, gradually decreased with higher pH values. This could be attributed to the absence of the drugs’ ionic forms. In order to ensure that MET and FEL were completely ionized and enable their simultaneous assessment by their respective electrodes, a pH value of 7.0 for MET and pH 3.0 for FEL was used.

#### Effect of soaking time

In this study, two electrodes, namely MET/MWCNTS and FEL-MIP/MWCNTS, were submerged in a solution with a concentration of 1 × 10^− 3^ M for varying durations, ranging from 1 h to 36 h. The purpose of this experiment was to investigate the effect of soaking time on the measured response. However, it is worth noting that the electro-active species leached into the soaking solution, which resulted in undesirable outcomes when the soaking duration exceeded 24 h [2]. Our research findings indicate that the optimal duration for achieving a slope of 55.230 and 52.365 mV/decade for MET/MWCNTS and FEL-MIP/MWCNTS, respectively, was determined to be 24 h of soaking.

#### Dynamic response time

For each drug concentration, the monitoring process involved recording the actual time required for the two electrodes to achieve a stable potential reading within ± 1 mV. Fig. S4 displays the potential/time curves for the proposed sensors, indicating a dynamic response time of approximately 5.0 s. In comparison to more traditional chromatographic procedures, ISEs are far more effective in drug quantification, as this rapid reaction showed.

#### Temperature effect

The potential behavior of MET/MWCNTS and FEL-MIP/MWCNTS was tested under varying temperatures between 25 and 35 °C using standard solutions of MET and FEL. Both sensors show very good thermal stability up to 35 °C, according to the recorded responses shown in Fig. S5 for each temperature. It is worth noting that higher temperatures have a destructive influence on such PVC-based membranes.

### Sensors selectivity

The (logK^pot^_D; I_), potentiometric selectivity coefficients were determined using the separate solution method (SSM) [39]. The two main electrodes, MET/MWCNTS and FEL-MIP/MWCNTS, were tested for their performance. The selectivity coefficients, which were found by measuring the 1 × 10^− 4^ M potential for each component, are summarized in Table 2. With no interference from inorganic ions like Na^+^, K^+^, or their degradation products, both electrodes showed a high level of selectivity towards the relevant medications. The results also showed that the two sensors might act as stability-indicating ISEs as the degradation products had poor selectivity coefficient values. With a logP value of 3.86 compared to MET’s logP value of 1.76, FEL is more lipophilic and could easily interact with the incorporated lipophilic ion exchanger within the sensing membrane. Fortunately, ionization of FEL only occurs at pH 2.0–4.0, hence it is possible to measure MET at higher pH value without any interference even though FEL’s lipophilicity presents a problem.

**Table 2:** Logarithmic selectivity coefficients, of the proposed electrodes

Interferent	log (K^pot^_MET, interferent_) ^a^	log (K^pot^_FEL, interferent_) ^b^
Met	0.00	-1.47
FEL	-2.23	0.00
MET degradate	-1.51	-1.79
FEL degradate	-2.43	-1.48
Na^+^	-2.53	-2.41
K^+^	-2.49	-2.55
Ca^+ 2^	-2.73	-2.93
Mg^+ 2^	-2.88	-3.01
Glycine	-3.15	-3.28
Starch	-3.22	-3.35
Sucrose	-3.28	-3.31
Microcrystalline cellulose	-3.30	-3.43

^a^ Average of three determinations

### Application to Logimax® and spiked human plasma

The newly developed sensors were employed to concurrently quantify MET and FEL in Logimax^®^ capsules, a recently introduced combination drug formulation. For each sensor, the concentration of the respective drug was derived from its potentiometric response based on a pre-determined regression equation. The mean percent recoveries achieved were 99.95 ± 1.687 for MET and 97.69 ± 1.485 for FEL, demonstrating high accuracy. Furthermore, the sensors were also tested in spiked human plasma samples, yielding mean recoveries of 98.55 ± 1.814 for MET and 97.06 ± 1.789 for FEL. These satisfactory outcomes underscore the efficacy of the sensors for direct, simultaneous measurement of MET and FEL, with no detectable interference from either compound, thereby highlighting their potential application in clinical and pharmaceutical analysis.

### Statistical comparison & method evaluation

To ensure the validity of the stated sensors in detecting MET and FEL, we employed a statistical comparison between the obtained results with those obtained using the official methods for MET [9] and FEL [12]. Table S1 shows that the calculated t- and F-values were lower than the tabular ones, providing strong evidence that the difference between the official procedures and the recommended ones is negligible. In addition, as shown in Table 3, comparative research was carried out to assess the performance of the suggested sensors in relation to other electrochemical works that have been reported for MET [25–28]. The proposed method showed better linearity range, lower limit of detection and wider application.

**Table 3:** An overview on the reported methods for the determination of MET

Ref. No.	Analyte	Linearity range	LOD	Application
25	MET	2.00 × 10^− 7^- 8.00 × 10^− 3^ mol L^− 1^	1.26 × 10^− 7^ mol L^− 1^	Single tablet, human plasma and urine samples
26	MET	1.00 × 10^− 6^ − 8.00 × 10^− 2^ mol L^− 1^	5.5 × 10^− 6^ mol L^− 1^	Tablet and human plasma
27	MET	2.00 × 10^− 7^ − 1.00 × 10^− 2^ mol L^− 1^	3.2 × 10^− 7^ mol L^− 1^	Pharmaceutical preparations
28	MET	1.00 × 10^− 5^ − 1.00 × 10^− 1^ mol L^− 1^	4.5 × 10^− 6^ mol L^− 1^	Pharmaceutical preparations
This work	MET	1.00 × 10^− 7^ − 1.00 × 10^− 2^ mol L^− 1^	5 × 10^− 8^ mol L^− 1^	Combined dosage form, human plasma and in presence of degradation products
	FEL	1.00 × 10^− 7^ − 1.00 × 10^− 4^ mol L^− 1^	7 × 10^− 8^ mol L^− 1^	

## Conclusion

Electrochemical methods currently show a significant role in pharmaceutical analysis. Despite the advantages of SC-ISEs, such as accuracy, sensitivity, rapidity, and reproducibility, the formation of a water layer remains a main disadvantage. MWCNTs have been previously utilized to avoid and overwhelm this issue. The present study presented a novel potentiometric method for quantifying two drugs with identical charges and lipophilic characteristics simultaneously. The method demonstrated its efficacy in resolving the nosiness that traditionally impeded the potentiometric assessment of MET and FEL. The suggested electrodes were able to determine each drug in-presence of their degradates, pharmaceutical tablets and spiked human plasma. The presented approach could be applied for the therapeutic drug monitoring of the studied drugs’ concentrations in body fluids. This study is also a useful attempt that may be exploited for further designing portable sensors to be employed in pharmacokinetics.

## Electronic supplementary material

Below is the link to the electronic supplementary material.

Supplementary Material 1

## Data Availability

The datasets used and/or analysed during the current study are available from the corresponding author on reasonable request.

## Abbreviations

MIP: Molecularly imprinted polymer
SC-ISEs: Solid contact ion-selective electrodes
CNTs: Carbon nanotubes
MWCNTs: Multi-walled carbon nanotubes
MET: Metoprolol Succinate
FEL: Felodipine
AIBN: Azobisisobutyronitrile
APS: Ammonium persulfate
DMSO: Dimethylsulfoxide
PVC: Polyvinyl chloride
SDS: Sodium dodecyl sulfate
EGDMA: Ethylene glycol dimethacrylate
THF: Tetrahydrofuran
MAA: Methacrylic acid
TpClPB: potassium tetrakis (4-chlorophenyl) borate
NPOE: 2-nitrophenyl octyl ether
BRB: A Britton-Robinson buffer
CPE: Carbon paste electrode
NIP: Non-imprinted polymer
FE-SEM: Field Emission-Surface Electron Microscopy
DSC: Differential scanning calorimetry
HPLC: High-performance liquid chromatography
HPTLC: High-performance thin-layer chromatography
SSM: Separate solution method
