# Supplementary material for: A stability-indicating potentiometric platform for assaying Metoprolol succinate and felodipine in their tablets and human plasma
Source: BMC Chem. 2025 Mar 19;19(1):73. doi: 10.1186/s13065-025-01435-z (PMC11924619; doi:10.1186/s13065-025-01435-z)
Supplement: Supplementary file 1 — Supplementary Material 1 [file 13065_2025_1435_MOESM1_ESM.docx]

Supplementary Material

for

**A stability-indicating potentiometric platform for assaying metoprolol succinate and felodipine in their tablets and human plasma**

Haitham A. El Fiky^a^, Mahmoud A. Tantawy^b, c*^, Dina A. Ahmed^a^, Maha F. Abd El Ghany^d^, Amr M. Badawey^b^, Nermine V. Fares^d^

^a^ Pharmaceutical Chemistry Department, Faculty of Pharmaceutical Sciences and Pharmaceutical Industries, Future University in Egypt, Cairo, Egypt.

^b^ Pharmaceutical Analytical Chemistry Department, Faculty of Pharmacy, Cairo University, Kasr El-Aini Street, ET-11562, Cairo, Egypt.

^c^ Department of Chemistry, Faculty of Pharmacy, October 6 University, 6 of October City, Giza, Egypt.

**^d^** Analytical Chemistry Department, Faculty of Pharmacy, Ain shams University, Cairo, Egypt.

^*^ E-mail address: [mahmoud.eltantawy@pharma.cu.edu.eg](mailto:mahmoud.eltantawy@pharma.cu.edu.eg)


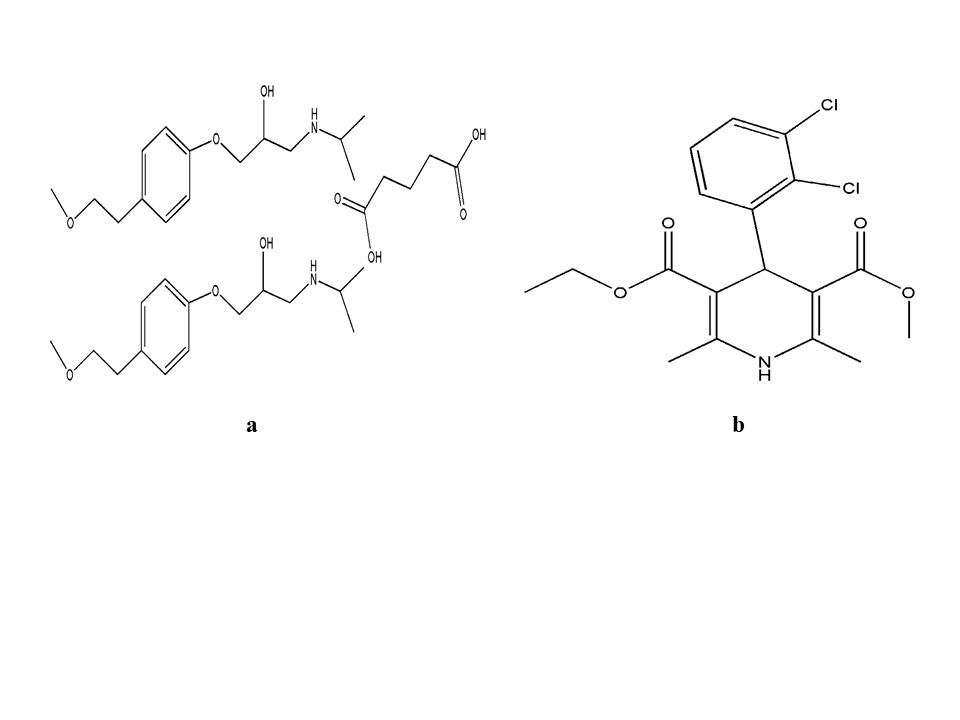


**Fig. S1**. Chemical structure of (a) metoprolol succinate and (b) felodipine.

**
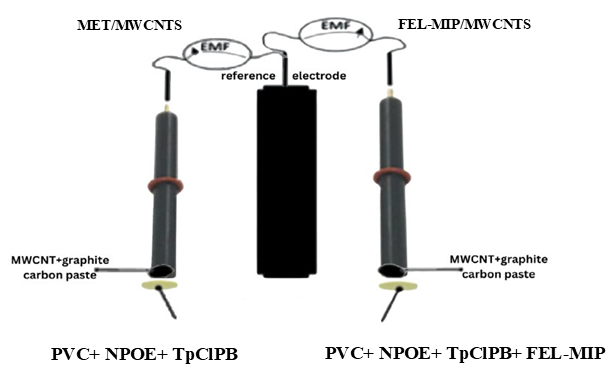
**

**Fig. S2**. An illustration of the proposed potentiometric system.

**
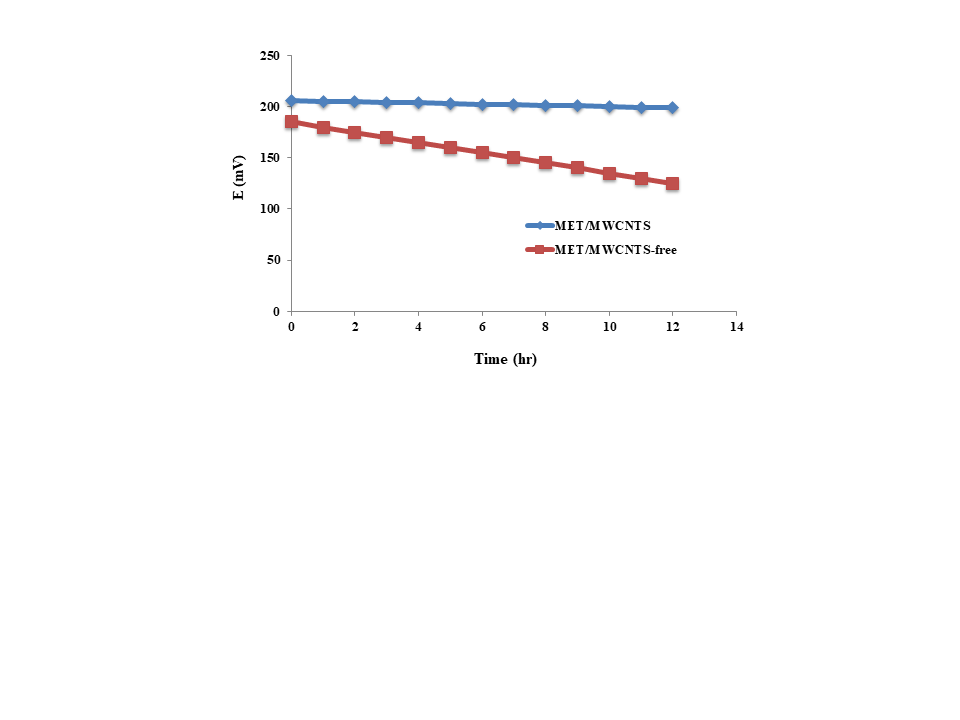
**

**Fig. S3**. Potential stability of the proposed sensors over 12 hours for a 1.0×10^-4^ mol L^−1^ solution of metoprolol.

**
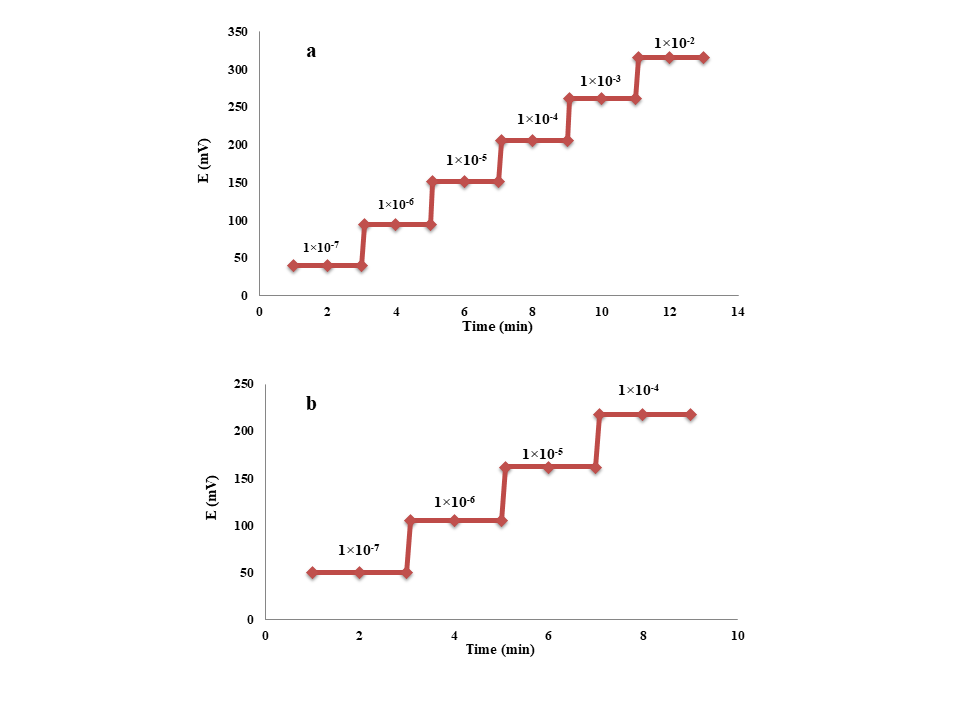
**

**Fig. S4**. Plot of potential as a function of time for responses of (a) MET/MWCNTS and (b) FEL-MIP/MWCNTS electrodes towards increasing concentrations of their solutions.

**
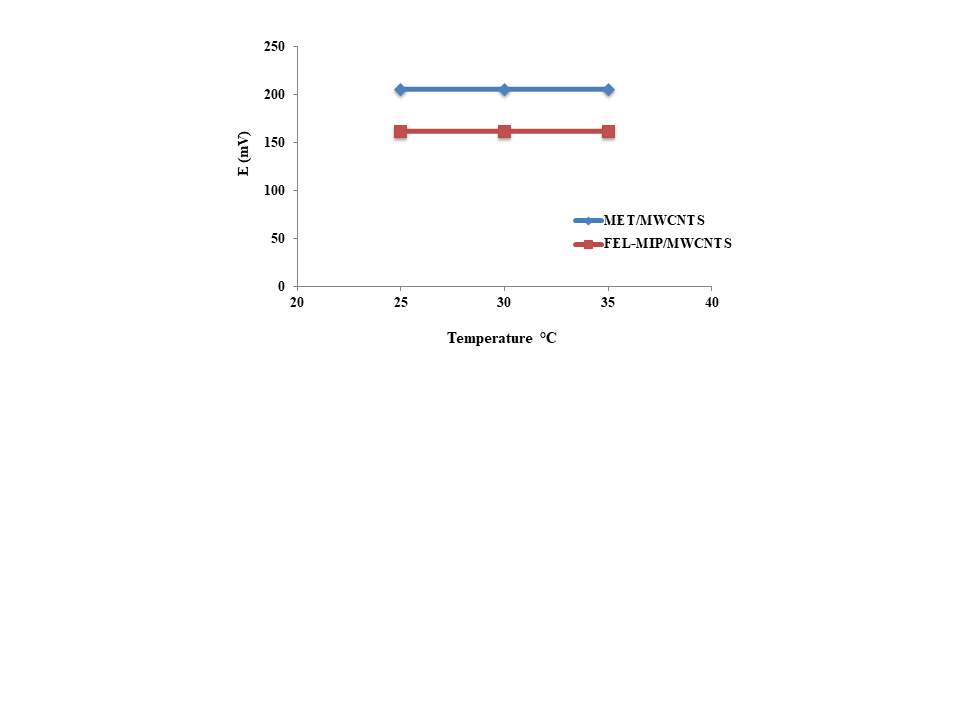
**

**Fig. S5**. Effect of Temperature on MET/MWCNTS and FEL-MIP/MWCNTS electrodes.

**Table S1**. Statistical comparison of obtained results and the official methods.

| **Parameter** | **MET** | **Official method^b^** | **FEL** | **Official method^c^** |
| --- | --- | --- | --- | --- |
|  |  |  |  |  |
| **Mean** | 99.97 | 99.55 | 100.67 | 99.65 |
| **SD** | 1.48 | 0.65 | 0.46 | 0.98 |
| **N** | 5 | 4 | 5 | 4 |
| **Variance** | 2.19 | 0.42 | 0.21 | 0.96 |
| **Student^'^s *t* test (2.365)^a^** | 0.57 | - | 1.919 | - |
| **F-test** | 5.214 **(15.10)^a^** | - | 4.571  **(9.98) ^a^** | - |

^a^ The values in parenthesis are the corresponding theoretical values of t and F at p = 0.05.

^b^ Official Potentiometric titration method for determination of Metoprolol succinate against perchloric acid**.**

^c^ Official Potentiometric titration method for determination of Felodipine against cerium sulphate**.**
